# The effects of dialysis modalities on the progression of coronary artery calcification in dialysis patients

**DOI:** 10.1186/s12882-020-01963-x

**Published:** 2020-07-25

**Authors:** Qingyu Niu, Huiping Zhao, Li Zuo, Mei Wang, Liangying Gan

**Affiliations:** Department of Nephrology, Peking University People’s Hospital, 11 Xizhimennan South Street, Xicheng District, Beijing, 100044 China

**Keywords:** Hemodialysis, Peritoneal dialysis, Coronary artery calcification, Vascular calcification

## Abstract

**Background:**

Hemodialysis (HD) tend to have more hemodynamic changes than peritoneal dialysis (PD), which aggravates inflammation and oxidative stress. Whether HD and PD have different effects on the progression of vascular calcification? Therefore, we produced a study to explore the relationship of dialysis modalities and coronary artery calcification (CAC) progression.

**Methods:**

This was a prospective cohort study. CT scans were performed at enrollment and 2 years later for each patient. Demographic and clinical data were collected. Tobit regression was used to compare delta CAC score between HD and PD patients.

**Results:**

(1) 155 patients were enrolled, including 69 HD and 86 PD patients. (2) The baseline CAC scores were 97 (1, 744) in HD and 95 (0, 324) in PD; the follow-up CAC scores were 343 (6, 1379) in HD and 293 (18, 997) in PD. There were no significant differences in baseline, follow-up and delta CAC scores between 2 groups (*P* > 0.05). (3) In Tobit regression, after adjusted for variables, there was no significant difference of CAC progression in HD and PD groups (*P* > 0.05). (4) Logistic regression showed that older age, diabetes and higher time-averaged serum phosphate (P) were associated with faster progression of CAC (*P* < 0.05), but there was no evidence that HD was associated with faster CAC progression compared with PD (*P* = 0.879).

**Conclusions:**

There was no evidence that different dialysis modalities have different effect on CAC progression. Old age, DM and higher time-averaged P were associated with fast CAC progression.

## Background

Cardiovascular death has long been the leading cause of death in chronic kidney disease (CKD) patients [1, 2]. Coronary artery calcification (CAC) is an important factor that increase the risk of cardiovascular disease, which is a common pathological manifestation due to mineral and bone disorder in CKD patients [3–5].

When CKD progresses to end stage renal disease (ESRD), kidney replacement therapy is often required. Hemodialysis (HD) and peritoneal dialysis (PD) are the most common dialysis modalities for ESRD patients. There was controversy about the effects of dialysis treatment modalities on the survival of ESRD patients [6, 7]. Compared with PD, HD may have greater hemodynamic change and hyperdynamic circulation induced by interdialytic fluid accumulation, rapid ultrafiltration and arteriovenous fistula [8, 9]. These hemodynamic changes may cause vascular endothelial cell dysfunction and initiation of oxidative stress in HD patients. In the study of Lilien et al., they confirmed that HD procedure induces further endothelial dysfunction in children with ESRD by measuring arterial flow-mediated dilation [10]. Meanwhile, inadequate dialyzer membrane biocompatibility aggravates inflammation and oxidative stress when the artificial materials contact with blood [11]. Oxidative stress and inflammation are also important factors that contribute to vascular calcification [12, 13]. Inversely, PD has little influence on hemodynamics and better preservation of residual renal function, so PD patients may possibly be at a lower cardiovascular risk [14].

However, whether these two dialysis modalities have different effects on the progression of vascular calcification is currently inconclusive. We therefore performed a prospective cohort study of patients with HD and PD to compare the effects of different dialysis modalities on the progression of CAC.

## Methods

### Study design and subjects

This is a prospective cohort study. We enrolled maintenance HD and PD patients with ESRD in our dialysis center from January 2012 to January 2015. Inclusion criteria: (1) age ≥ 18 years old; (2) dialysis vintage ≥3 months; (3) with stable clinical condition. Exclusion criteria: (1) conditions making CT technically impossible or unreliable (such as severe cardiac arrhythmias); (2) patients who are pregnant or plan to become pregnant within 2 years; (3) patients with acute complications such as heart failure, severe infection, malignant tumor and life expectancy less than 3 months.

### Demographic and clinical data

Baseline demographics were collected, including age, gender, dialysis vintage, primary causes of ESRD, diabetes mellitus (yes or no), body mass index (BMI) and the history of cardiovascular disease (yes or no). Laboratory indices were tested every 3 months during the follow-up period, then calculated the time-averaged values, including serum corrected calcium (cCa), phosphate (P), serum intact parathyroid hormone (iPTH), serum albumin (Alb), serum creatinine (Scr), hemoglobin (Hb), serum triglycerides (TG), low-density lipoprotein cholesterol (LDL-C), high-density lipoprotein cholesterol (HDL-C), total cholesterol (T-Cho) and serum C-reactive protein (CRP). Relative medication use was collected, including calcium-based phosphate binder, non-calucium-based phosphate binder, cinacalcet and vitamin D analogue.

### Evaluation of coronary artery calcification

CT scans were performed at enrollment and 2-years later in Department of Radiology of our hospital, CAC scores assessed blindly by two radiologists according to the method previously described by Agaston et al. [15]. Delta (∆) CAC score was defined as the absolute difference between follow-up CAC score and baseline CAC score, reflecting the progression of CAC during the two-year follow-up period. To analyze the risk factors of CAC progression, subjects were also classified as ∆CAC score ≤ 100 and ∆CAC score > 100.

### Statistical methods

Continuous variables were expressed as mean ± standard deviation or median with 25th–75th percentile, and categorical data were expressed as number and percentage. Differences in variables between groups (HD vs. PD; ∆CAC score ≤ 100 vs. > 100) were evaluated by using independent sample t-test or the Wilcoxon rank-sum test on the basis of whether the data were normally distributed. Categorical variables of groups were compared using chi-square test. Changes in CAC scores from baseline to the end of follow-up in each group were compared by paired sample t-test. We compared the differences of ∆CAC score between two groups by using the Wilcoxon rank-sum test. In the univariate analysis of group ∆CAC score ≤ 100 and > 100, variables with *P* < 0.100 were included into multivariate logistic regression model to explore the independent risk factors of CAC progression. Also, important covariates know to be associated with vascular calcification based on clinical insight, such as serum cCa, P, iPTH were retained in the multivariate logistic regression model.

We evaluated ∆ CAC score between groups with Tobit regression as it was suitable to analyze variables with floor or ceiling effects as described in previous studies [16]. In our study, there were several patients have no detectable CAC at baseline and still no CAC at the end of 2-year follow up, so ∆ CAC scores of these patients were 0. In Tobit regression, we can assume that the endpoint variables (∆ CAC score) is a normally distributed variable that has been truncated by value of 0. We made 1 unadjusted model and 4 adjusted models in Tobit regression. The adjusted covariates included in model 1: age, gender; model 2: model 1 variables plus dialysis vintage and diabetes; model 3: model 2 variables plus serum Alb, cCa, P, iPTH, CRP and T-cho; model4: model 3 variables plus non-calcium P binder and Vitamin D analogue. *P* value < 0.05 was considered to be statistically significant. Tobit regression was performed by STATA software, version14.0, and other statistical analyses were performed using SPSS software, version 22.0. And figures were produced by PRISM software, version 6.0.

## Results

### Demographic data and clinical characteristics

We initially enrolled 155 patients, including 69 HD patients and 86 PD patients. All of them were performed CT test at the enrollment. After 2 years, there were 120 patients (57 in HD, 63 in PD) finished the follow-up CT test. Reasons of elimination including kidney transplantation, transferation, death, motion artefacts or stents of CT scans, and bypass surgery. In HD group, primary causes of ESRD were predominantly chronic glomerulonephritis (*n* = 38, 55.1%), followed by diabetic nephropathy (*n* = 14, 20.2%), chronic tubulointerstitial nephropathy (*n* = 6, 8.7%), hypertensive nephropathy (*n* = 4, 5.8%), and others (*n* = 7, 10.1%); in PD group, there were chronic glomerulonephritis (*n* = 40, 46.5%), diabetic nephropathy (*n* = 22, 25.6%), hypertensive nephropathy (*n* = 12, 14.0%), chronic tubulointerstitial nephropathy (*n* = 8, 9.3%), and others (n = 4, 4.7%) (Table 1).

**Table 1:** Characteristics of patients on hemodialysis or peritoneal dialysis

	HD (*n* = 69)	PD (*n* = 86)	*P* Value
Age (years)	52.1 ± 13.3	54.2 ± 11.7	0.306
Dialysis vintage (months)	38.0 (12.0,75.0)	26.0 (12.8,58.0)	0.084
Male (n, %)	47 (68.1)	39 (45.4)	0.006*
DM (n, %)	17 (24.6)	36 (41.9)	0.028*
**Primary causes of ESRD [n (%)]**			
CGN	38 (55.1)	40 (46.5)	0.289
DN	14 (20.3)	22 (25.6)	0.438
CTIN	6 (8.7)	8 (9.3)	0.896
HN	4 (5.8)	12 (14.0)	0.107
Others	7 (10.1)	4 (4.6)	0.294
CVD in history, n (%)	19 (27.5)	21 (24.4)	0.621
BMI (Kg/m^2^)	22.64 ± 3.65	23.06 ± 3.60	0.476
**Time-averaged test value**			
Hb (g/L)	114.5 (111.0,116.4)	115.6 (110.9117.3)	0.345
Alb (g/L)	40.1 ± 2.3	38.3 ± 2.9	< 0.001*
cCa (mmol/L)	2.31 ± 0.34	2.38 ± 0.11	0.114
P (mmol/L)	1.66 ± 0.37	1.56 ± 0.62	0.335
iPTH (pg/ml)	170.6 (81.8363.2)	187.9 (107.8340.8)	0.615
Scr (umol/L)	1043 ± 227	960 ± 273	0.078
CRP (mg/L)	3.16 (1.53, 6.33)	2.44 (1.54, 4.53)	0.669
LDL-C (mmol/L)	2.13 ± 0.58	2.97 ± 0.74	< 0.001*
HDL-C (mmol/L)	0.92 (0.85, 1.09)	1.06 (0.93, 1.28)	0.006*
T-Cho (mmol/L)	4.24 ± 0.81	5.17 ± 0.96	< 0.001*
CRP (mg/L)	3.16 (1.53, 6.33)	2.44 (1.54, 4.53)	0.669
**Medication use**			
Calcium-based P binder^a^ (n, %)	56 (100.0)	56 (88.9)	0.029*
Non-calcium-based P binder^b^ (n, %)	3 (5.3)	12 (19.1)	0.023*
Cinacalcet (n, %)	2 (3.5)	1 (1.6)	0.918
Vitamin D analogue (n, %)	29 (50.9)	15 (23.8)	0.002*
Baseline CAC Score	97 (1744)	95 (0, 324)	0.361

*P < 0.05 Notes: CGN: chronic glomerulonephritis; DN: diabetic nephropathy; CTIN: chronic tubulointerstitial nephropathy; HN: hypertensive nephropathy; BMI: body mass index; Hb: hemoglobin; Alb: albumin; cCa: corrected calcium; P: phosphate; iPTH: Intact Parathyroid Hormone; ALP: alkaline phosphatase; CO_2_CP: carbon dioxide combining power; Scr: serum creatinine; UA: uric acid; TG: triglyceride; LDL-C: low density lipoprotein cholesterol; HDL-C: high density lipoprotein cholesterol; T-Cho: total cholesterol; a, calcium carbonate; b, lanthanum carbonate or sevelamer

In baseline, the mean age of HD group was 52.1 ± 13.3 years, 68.1% were male, median dialysis vintage was 38 (12, 75) months and 24.6% had diabetes mellitus (DM); and in PD group, the mean age was 54.2 ± 11.7 years, 45.4% were male, median dialysis vintage was 26 (12.8, 58.0) months and 41.9% had DM. Compared with HD patients, patients in PD group have higher proportions of female and DM, higher levels of time-averaged serum LDL-C, HDL-C and T-Cho, and lower levels of time-averaged serum Alb (Table 1).

### Coronary artery calcification

**Fig. 1:**
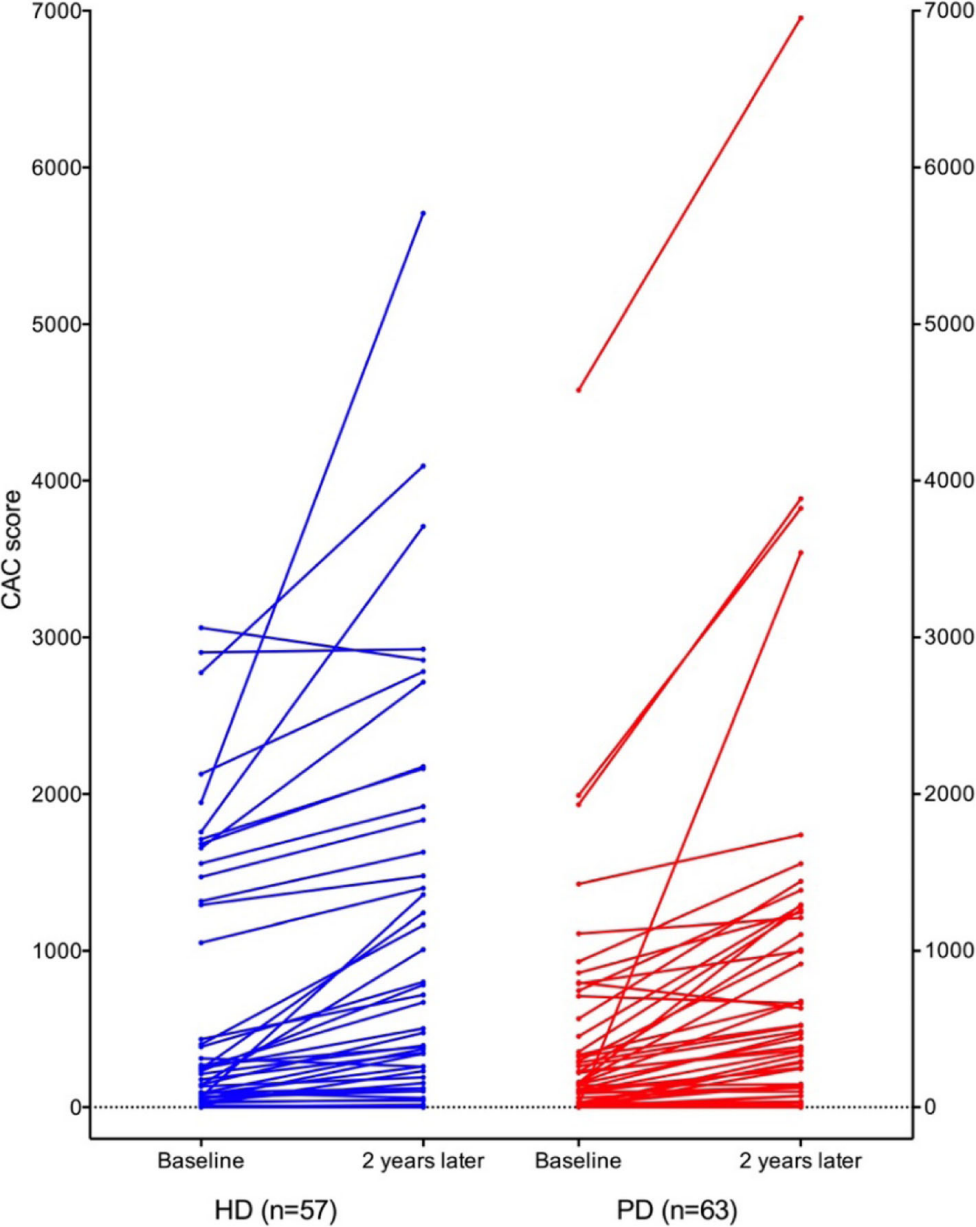
the progression of CAC in two groups

**Fig. 2:**
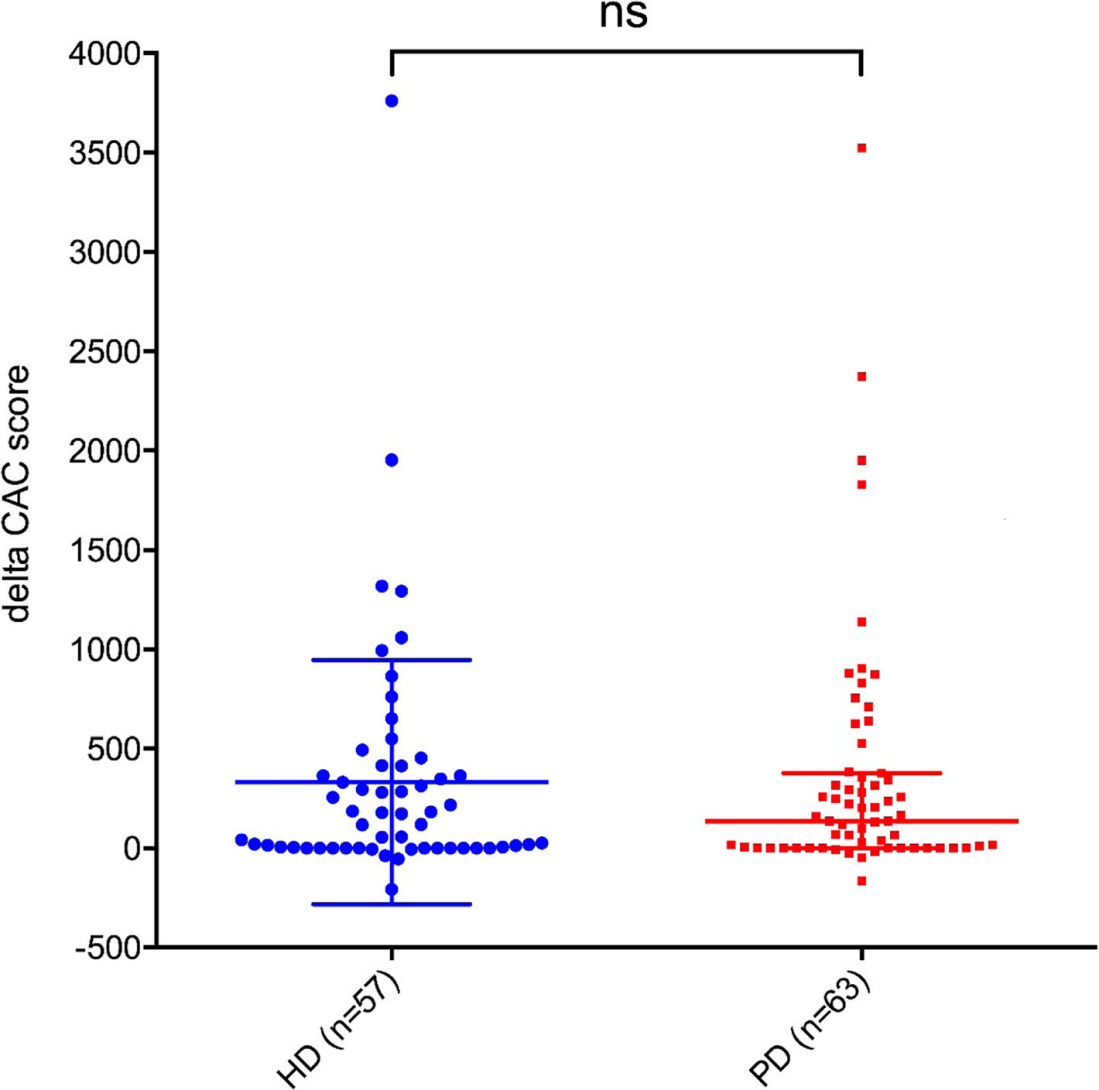
the delta CAC scores in two groups. (notes: Each point represented the increased value in coronary artery calcification scores during 2-year follow-up period of a patient)

The median of baseline CAC score in HD group was 97 (1744), and 95 (0,324) in PD group (Table 1). There was no significant difference (*P* = 0.361) in the baseline CAC score between 2 groups. Compared with baseline, CAC score of each group showed significant progress after 2-year follow-up (Table 2). But between the 2 groups, there was no significant difference in ∆CAC score: the median ∆CAC score in HD group was 119 (0, 389), and 136 (1, 377) in PD group (*P* = 0.766) (Table 2). In Fig. 1, we stratified patients by dialysis modality, and depicted the baseline CAC score and the progression trend of each patient as individual trajectories of CAC scores. And we compared ∆CAC scores between HD and PD patients in Fig. 2.

**Table 2:** Baseline and follow-up CAC scores and delta CAC scores of the two groups

	HD		P	PD		P
	Baseline (n = 69)	Month 24 (*n* = 57)		Baseline (n = 86)	Month 24 (*n* = 63)	
CAC score (mean ± SD)	538 ± 836	870 ± 1228	< 0.001*	335 ± 699	698 ± 1174	< 0.001*
CAC score median (25th,75th)	97 (1744)	343 (61379)	< 0.001*	95 (0,324)	293 (18,997)	< 0.001*
∆CAC score (mean ± SD)	332 ± 615			363 ± 633		0.788
∆CAC score median (25th, 75th)	119 (0, 389)			136 (1, 377)		0.766

Note: *P < 0.05

In Tobit regression, CAC score progressed with 92.17 per year in HD patients (95% CI: − 16.01 to 200.37) and with 126.80 per year in PD patients (95% CI: 28.54 to 225.07). In unadjusted model of Tobit regression, HD was not significantly associated with higher CAC progression comparing with PD (unadjusted difference: − 32.73 per year; 95% CI: − 174.9 to 109.4; *P* = 0.649). We performed 4 adjusted models in this part. When fully adjusted for age, gender, dialysis vintage, DM, Alb, cCa, P, iPTH, CRP and T-Cho, HD was also not significantly associated with faster progression of CAC than PD (adjusted difference: − 130.2 per year; 95% CI: − 308.0 to 47.5; *P* = 0.148) (Table 3).

**Table 3:** Effect estimates of CAC progression for PD compared with HD assessed by Tobit regression

∆CAC score (per year)	Coefficient	95% CI	P
Unadjusted model (PD as ref.)	−32.7	−174.9 to 109.4	0.649
Adjusted model 1 (PD as ref.)	−59.4	− 202.6 to 83.8	0.413
Adjusted model 2 (PD as ref.)	−44.7	−184.0 to 94.6	0.526
Adjusted model 3 (PD as ref.)	−112.3	− 285.7 to 61.2	0.201
Adjusted model 4 (PD as ref.)	−130.2	−308.0 to 47.5	0.148

Notes:

Adjusted model 1: adjusted for age and gender;

Adjusted model 2: adjusted model 1 + dialysis vintage and diabetes mellitus;

Adjusted model 3: adjusted model 2 + Alb, Ca, P, iPTH, CRP, T-Cho;

Adjusted model 4: adjusted model 3 + non-calcium P binder, Vitamin D analogue

### Subgroup analysis

Supplementary Table 1 summarized results of subgroup analyses for different conditions of CAC progression in HD and PD patients. CAC progressed significantly faster in patients with DM than in patients without DM, which can be seen in both HD and PD groups (in HD group, ∆CAC scores of patients with DM and without DM were 415 (198, 931) and 24 (0, 292) respectively, *P* = 0.004; in PD group, these were 280 (118, 734) and 25 (0, 278), respectively, *P* = 0.006;). But no significant difference of CAC progression between HD and PD groups (*P* > 0.05). For patients with dialysis vintage≤60 or > 60 months, there weren’t significant differences of CAC progression in both HD and PD patients (Supplement Table 1, *P* > 0.05). For HD group, older patients (age > 55) tend to has faster progression of CAC than younger patients (∆CAC scores in older patients were 172 (0, 474) and 51 (0, 347) in younger patients, *P* = 0.040). However, the different speeds of calcification progression in different age groups weren’t seen in PD patients, and there also no significant different between HD and PD groups (P > 0.05).

### Influencing factors of CAC progression

To explore the factors that influence the progression of CAC, we devided patients into 2 gourps: fast progression of CAC (∆CAC score > 100) and slow progression of CAC (∆CAC score ≤ 100). Compared with the slow progression group, patients with fast CAC progression exhibited older age, higher proportion of DM and use of calcium-based phosphate binder, higher BMI, time-averaged CRP (*P* < 0.05; Supplemental Table 2). In Logistic regression, after adjusted for confounders, the result showed that older age, DM and higher time-averaged serum P were independent risk factors of fast CAC progression (P < 0.05; Table 4), but no evidence shown that dialysis modality was associated with faster CAC progression (OR = 1.097, 95%CI: 0.331–3.634, *P* = 0.879, Table 4).

**Table 4:** Influencing factors of fast CAC progression in logistic regression model

Variables	B	OR	95%CI	P
Age (/per year)	0.045	1.046	1.000–1.095	0.049*
DM (yes vs. no)	1.394	7.714	2.095–28.402	0.002*
BMI (/per 1 Kg/m^2^)	0.121	1.129	0.946–1.347	0.179
Dialysis modalities (HD vs. PD)	0.093	1.097	0.331–3.634	0.879
Time-averaged Ca (/per 1 mmol/L)	−2.683	0.068	0.001–5.348	0.228
Time-averaged P (/per 1 mmol/L)	2.574	17.147	2.863–102.709	0.002*
Time-averaged iPTH (/per 1 mmol/L)	0.001	1.001	0.998–1.004	0.652
Time-averaged CRP (/per 1 mmol/L)	0.056	1.057	0.929–1.203	0.397

Note: *P < 0.05

## Discussion

Our study indicated whether dialysis modalities affect the progression of coronary calcification. In this prospective cohort, we enrolled 69 HD patients and 86 PD patients. After 2-year follow-up period, we didn’t find the significant differences of CAC progression between HD and PD groups. And in our study, older age, DM and higher time-averaged serum P were associated with faster CAC progression.

There were few studies have investigated the relationship of dialysis modality and the progression of vascular calcification. In Lee’s study [17], they included 15 PD patients and 18 HD patients who were tested for CAC scores at 1, 6 and 12 months. They didn’t find differences in CAC score between HD and PD patients. In the study of Jansz et al. [18], they enrolled 94 HD patients and 40 PD patients at baseline, but only 34 HD patients and 23 PD patients finished the 3-year follow-up period. Their results shown that patients on PD do not have less CAC progression than patients on HD. However, the sample size of these studies was small.

According to literatures, it was reasonable if we confirm a hypothesis that PD patients have less progression of vascular calcification than HD patients, but our results were negative. There were several reasons that we got these negative results. First, in our dialysis center, the proportion of diabetes in PD patients was significantly higher than that in HD patients, which contribute a lot to the occurrence and progression of vascular calcification. Second, it can be seen from the clinical and laboratory data that the nutritional status of PD patients was worse than that of HD patients (such as serum Alb). Then, the disorders of lipid metabolism in PD patients was more severe. In other words, the overall condition of our PD patients was worse than that of HD patients. Therefore, it was expected that the prevalence of CAC is higher, and the progression is faster in our PD patients comparing to HD group. However, our results did not show a faster progression of CAC in the PD group.

We also analysed the independent risk factors of fast CAC progression by using logistic regression model. After adjusted for covariates, the result showed that older age, DM, higher time-averaged serum P were independent risk factors of fast CAC progression. Aging and diabetes are recognized risk factors for the occurrence and progression of vascular calcification, and this was reported by many studies before [19–21]. Meanwhile, among various risk factors of vascular calcification in CKD patients, hyperphosphatemia is most strongly involved with calcification and a main part of CKD-MBD [22]. Many clinical researches have investigated that hyperphosphatemia is nearly associated with advanced vascular calcification [22–25]. And in vitro studies, high-phosphorus medium can calcify vascular smooth muscle cells [26]; in vivo studies, Pi loading can promote vascular calcification in uremic rodents [27]. However, in our study, we didn’t find the relationship between serum Ca and iPTH levels and progression of CAC. The possible reason may be that our center has strictly adhered to continuous quality improvement for Ca and P metabolic disorders, so laboratory data of most patients were within the optimal range, which minimizes the risk of complications and mortality in patients [28].

There were several strengths of our study. First, till now, our study was the largest to compare the progression of vascular calcification between different dialysis modalities. Second, among patients we included, the rate of loss of follow-up was relatively low, ensuring the stability of the results. Third, we chose the tobit regression to analysis the different of ∆CAC score between HD and PD patients. The Tobit model, also called a censored regression model, is designed to estimate linear relationships between variables when there is either left- or right-censoring in the dependent variable, which has been used in many areas of medical science [29, 30]. However, there were also some limitations. First, there were differences in the clinical situation of patients between HD and PD groups, which may affect the effect of comparison. Meanwhile, the follow-up period wasn’t long enough. Since vascular calcification progresses slowly, it may take longer to detect changes.

## Conclusions

In summary, we did’n find the significant different effects between HD and PD on CAC progression. This indicated that PD may not associated with less vascular calcification progression. This result may provide some clinical evidence for choosing the appropriate dialysis modalities. And in our study, older age, DM, higer time-averaged serum P were associated withfast CAC progression. Large sample size and high-quality clinical research is still needed in the future to explore the effects of different dialysis modalities on vascular calcification.

## Supplementary information

**Additional file 1.**

## Data Availability

The data used of this study are available from the corresponding author on reasonable request.

## Abbreviations

CKD: chronic kidney disease
ESRD: end stage renal disease
CVD: cardiovascular disease
CAC: coronary artery calcification
HD: hemodialysis
PD: peritoneal dialysis
CT: computed tomography
BMI: body mass index
cCa: corrected calcium
P: phosphate
iPTH: serum intact parathyroid hormone
ALB: albumin
TG: triglycerides
LDL-C: low-density lipoprotein cholesterol
HDL-C: high-density lipoprotein cholesterol
T-Cho: total cholesterol
Scr: serum creatinine
Hgb: hemoglobin
