# Supplementary material for: The effects of dialysis modalities on the progression of coronary artery calcification in dialysis patients
Source: BMC Nephrol. 2020 Jul 25;21:302. doi: 10.1186/s12882-020-01963-x (PMC7382852; doi:10.1186/s12882-020-01963-x)
Supplement: Supplementary file 1 — Additional file 1. [file 12882_2020_1963_MOESM1_ESM.docx]

Supplemental table 1. Subgroup analysis of delta CAC score in HD and PD groups

|  |  | Detla CAC score | |  |
| --- | --- | --- | --- | --- |
|  |  | HD (n=57) | PD (n=63) | P  (HD vs. PD) |
| No DM | | 24 (0, 292) | 25 (0, 278) | 0.970 |
| DM | | 415 (198, 931) | 280 (118, 734) | 0.361 |
| P (no DM vs. DM) | | 0.004* | 0.006* |  |
| Vintage≤60 | | 19 (0, 265) | 162 (1, 326) | 0.185 |
| Vintage>60 | | 284 (17, 553) | 119 (9, 619) | 0.680 |
| P (Vintage≤60 vs. >60) | | 0.654 | 0.798 |  |
| Age≤55 | | 51 (0, 347) | 119 (5, 306) | 0.675 |
| Age>55 | | 172 (0, 474) | 158 (12, 385) | 0.387 |
| P (Age≤55 vs. >55) | | 0.040* | 0.418 |  |

Supplemental table 2. Compare the clinical characteristics of groups with fast and slow CAC progression

|  | Delta CAC score≤100  (n=55) | | Delta CAC score>100  (n=65) | | P | |
| --- | --- | --- | --- | --- | --- | --- |
| Age (years) | | 51.9±14.9 | | 57.4±9.7 | | 0.021* |
| Dialysis vintage (months) | | 53 (36, 86) | | 56 (38, 91) | | 0.718 |
| Male (n, %) | | 27 (49.1) | | 41 (63.1) | | 0.123 |
| DM (n, %) | | 8 (14.5) | | 30 (46.2) | | <0.001* |
| CVD in history (n, %) | | 9 (16.4) | | 19 (29.3) | | 0.108 |
| BMI（kg/m^2^） | | 21.6±3.2 | | 23.4±3.5 | | 0.004* |
| Hb (g/L) | | 115.0±8.5 | | 113.8±6.9 | | 0.388 |
| Alb (g/L) | | 39.5±3.0 | | 38.8±2.6 | | 0.147 |
| cCa (mmol/L) | | 2.34±0.34 | | 2.36±0.15 | | 0.720 |
| P (mmol/L) | | 1.53 (1.33, 1.74) | | 1.62 (1.41, 1.82) | | 0.072 |
| iPTH (pg/ml) | | 172 (95, 350) | | 189 (94, 387) | | 0.775 |
| Scr (umol/L) | | 1005±248 | | 995±262 | | 0.831 |
| UA (umol/L) | | 405±61 | | 413±64 | | 0.456 |
| TG (mmol/L) | | 1.81 (1.36, 2.52) | | 2.00 (1.53, 2.63) | | 0.212 |
| LDL-C (mmol/L) | | 2.53±0.78 | | 2.61±0.80 | | 0.591 |
| HDL-C (mmol/L) | | 1.04 (0.91, 1.21) | | 0.96 (0.86,1.15) | | 0.131 |
| T-Cho (mmol/L) | | 4.65 (3.92, 5.41) | | 4.58 (4.08, 5.35) | | 0.701 |
| CRP (mg/L) | | 1.86 (1.04, 4.13) | | 4.22 (1.71, 7.57) | | 0.008* |
| **Medication use** | |  | |  | |  |
| Calcium-based phosphate binder^a^ (n, %) | | 48 (87.3) | | 64 (98.5) | | 0.046* |
| Non-calcium-based phosphate binder^b^ (n, %) | | 5 (9.1) | | 10 (15.4) | | 0.299 |
| Cinacalcet (n, %) | | 0 (0) | | 3 (4.6) | | 0.312 |
| Vitamin D analogue (n, %) | | 20 (36.4) | | 24 (36.9) | | 0.990 |
